# CNS and CNS diseases in relation to their immune system

**DOI:** 10.3389/fimmu.2022.1063928

**Published:** 2022-11-16

**Authors:** Jianhao Xu, Canyu Ma, Menglu Hua, Jiarui Li, Ze Xiang, Jian Wu

**Affiliations:** ^1^ Department of Laboratory Medicine, The Yangzhou University Jianhu Clinical College, Jianhu, China; ^2^ Zhejiang University School of Medicine, Hangzhou, Zhejiang, China; ^3^ Department of Clinical Laboratory, The Affiliated Suzhou Hospital of Nanjing Medical University, Suzhou Municipal Hospital, Gusu School, Nanjing Medical University, Suzhou, Jiangsu, China

**Keywords:** central nervous system (CNS) injury, immunocytes, immune system, neuro-immune interaction, role

## Abstract

The central nervous system is the most important nervous system in vertebrates, which is responsible for transmitting information to the peripheral nervous system and controlling the body’s activities. It mainly consists of the brain and spinal cord, which contains rich of neurons, the precision of the neural structures susceptible to damage from the outside world and from the internal factors of inflammation infection, leading to a series of central nervous system diseases, such as traumatic brain injury, nerve inflammation, etc., these diseases may cause irreversible damage on the central nervous or lead to subsequent chronic lesions. After disease or injury, the immune system of the central nervous system will play a role, releasing cytokines to recruit immune cells to enter, and the immune cells will differentiate according to the location and degree of the lesion, and become specific immune cells with different functions, recognize and phagocytose inflammatory factors, and repair the damaged neural structure. However, if the response of these immune cells is not suppressed, the overexpression of some genes can cause further damage to the central nervous system. There is a need to understand the molecular mechanisms by which these immune cells work, and this information may lead to immunotherapies that target certain diseases and avoid over-activation of immune cells. In this review, we summarized several immune cells that mainly play a role in the central nervous system and their roles, and also explained the response process of the immune system in the process of some common neurological diseases, which may provide new insights into the central nervous system.

## Introduction

The immune processes involved in these two types of immunity and the therapeutic regulation of disease and injury by immune cells are not unidirectional in their effects on the CNS, for the reason that the wrong activation or over-response can cause damage to the central nervous system. The developed nervous system of vertebrates is an important sign of high evolution (1, 2). Central Nervous System (CNS) is the main component of the human nervous system, including the brain, which is located in the skull cavity, and the spinal cord, which is located in the spinal canal. CNS can accept incoming information from all over the body, integrate and process it, and finally control the body through outgoing signals (3), or use this information to complete memory and learning, so that the organism can carry out a series of thinking activities.

Because the CNS is the most important vertebrate nervous system, and the brain and spinal cord, the two main components of the CNS, have been shown to be non-renewable (4). Therefore, CNS injury and disease and the subsequent repair of immune responses are crucial for the CNS. Like the immune system of most other tissues, the immune system of CNS is composed of the innate immune system and the specific immune system (5, 6). The innate immune system is mainly composed of congenital macrophages. The main process of specific immunity is the specific immune response produced by the specific combination of antigens and antibodies. The immune process involved in these two immune modes and the therapeutic regulation of immune cells on diseases and injuries have a non-unidirectional effect on the CNS —— incorrect activation or excessive response may cause damage to the central nervous system (7).

At present, there is a relatively clear understanding of the birth and differentiation of immune cells, and more understanding of the possible functions of most immune cells after differentiation. Studies on CNS immunity from 2020 to the present have mainly focused on the molecular mechanism of immune cells entering the CNS and promoting CNS inflammation, and on this basis, how to regulate the response degree of immune cells. For some immune cells, the researchers found other types of differentiation and other effects. Other studies have attempted to explain the link between CNS immunity and other diseases, and have attempted to use CNS immunotherapy as a clinical treatment (8, 9).

In this review, we illustrated the immune components of CNS and their roles, observe the performance of these immune components in different neurological diseases, and judged whether they are beneficial or harmful for CNS diseases. Based on this information, we may find ideas and methods of immunotherapy for these diseases.

## Central nervous system

### Composition of CNS

**Figure 1:**
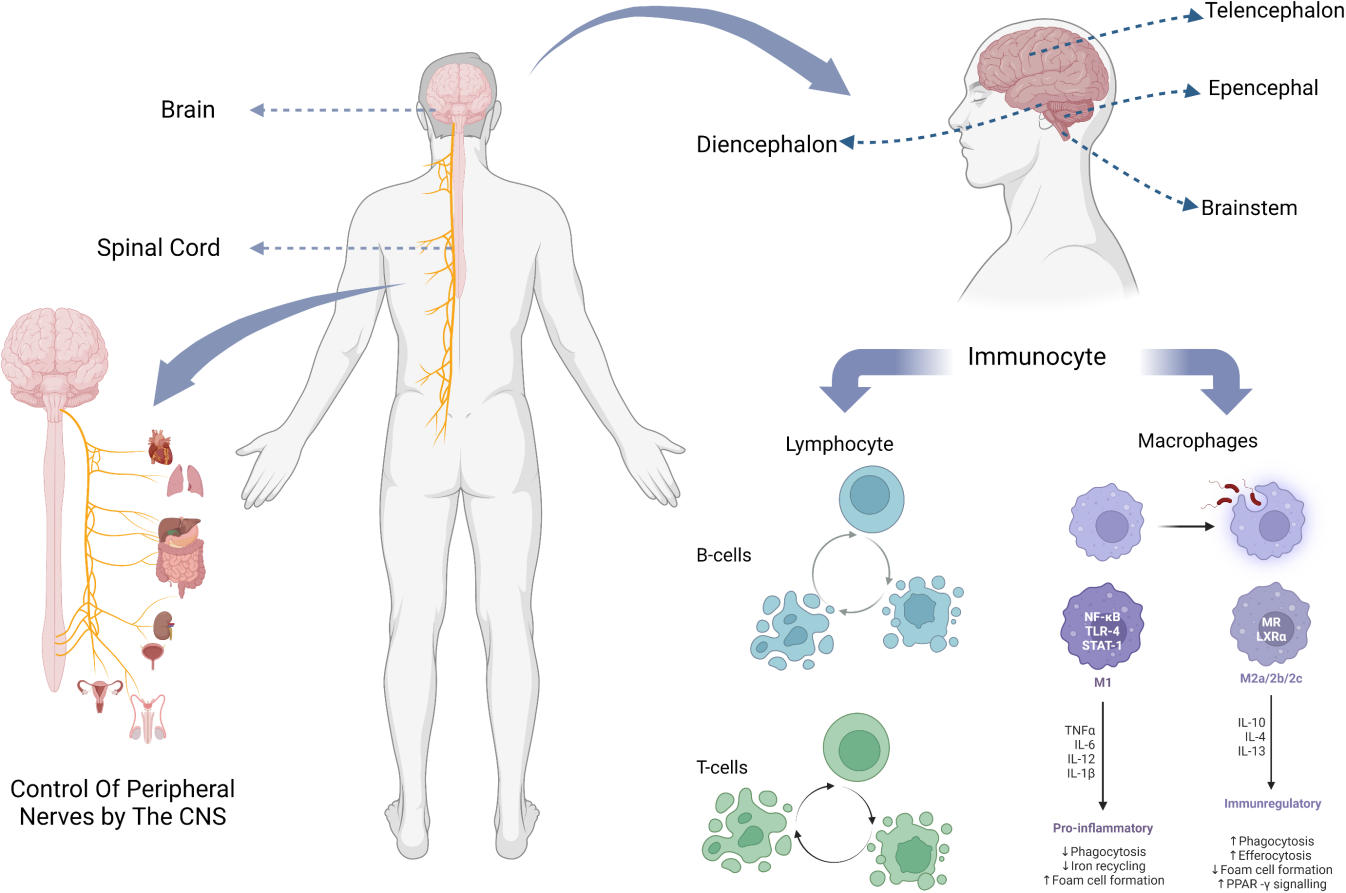
The composition of the CNS, the location of its parts, and the major immune cells it contains.

The central nervous system of the human body consists of the brain and the spinal cord (**Figure 1**). The spinal cord is the lowest level of motor center, and it is also the basic reflex center to complete the movement of the organism, under the control of the brain, through the neural circuit signal conduction, so as to achieve motor regulation. The spinal cord is composed of gray and white matter and contains nerve cell bodies and their ascending and descending conduction tracts (10). Classical studies of spinal cord function have focused on well-defined neural pathways that are thought to mediate automatic functions of stereotyping, such as stretch and flexion reflexes, and allocate inputs from sensory and descending fibers to appropriate targets (11).

Brain, generally refers to three parts which are: cerebellum, brainstem, diencephalon. The brainstem is an important part of the brain responsible for sensing injury and processing pain signals, and transmits and processes signals between the brain, cerebellum and spinal. The conventional wisdom about the cerebellum is that it is essential for motor function and contributes little to cognitive function (12). However, multiple studies by Middleton FA et al. showed that cerebellar output targets involved multiple cortical regions, including not only primary motor cortex (13, 14), but also oculomotor nerve (15, 16), prefrontal lobe and inferior temporal region (17, 18), which indicates that cerebellum plays a role in both motor and cognitive function.

### Working mechanism of CNS

The sensory organs with receptors establish communication with the central nervous system through the afferent nerves, so as to realize the regulation of the central nervous system. Peripheral nerve tissue is composed of afferent sensory fibers and efferent motor fibers. When receptors such as the skin are stimulated by the outside world, they will produce nerve impulses. Nerve synapses release neurotransmitters, which are received by cells, and the resting potential is converted into action potential (19). After receiving the neurotransmitter, the receiver may process it and react on the afferent nerve synapse (20), and intercellular conduction and closed interaction of nerve electrical signals are realized. The electrical signal is transmitted to the CNS along the sensory fibers, and the CNS processes the signal and transmits it along the motor fibers to control the body movement (21). In this way, the CNS is connected to the rest of the nervous system.

Take, for example, the mechanism of regulation of renal afferent sensory nerves in the central nervous system. Kidney is an important sensory organ with abundant baroreceptors and chemoreceptors, as well as a large number of afferent nerves. Renal afferents can directly project to areas of the central nervous system, such as the lateral thalamic area and the paraventricular nucleus (PVN), and indirectly project to other areas of the hypothalamus (22). During stimulation of the afferent renal nerve, the firing frequency of the large-cell neurons in the PVN increased (23). Renal afferents activate the central nervous system and enhance sympathetic activity (24).

In simple terms, the peripheral nervous system gives information to the CNS in the form of electrical stimulation through the afferent nerve, which is processed by the CNS to realize the regulation of the body.

## Immune cells and immune system in the CNS

### Immune cells

Among all immune cells in the central nervous system, macrophages and glial cells derived from them, as well as lymphocytes including T cells, B cells and self-killing immune cells, play a major role (**Table 1**).

**Table 1:** The distribution, roles, types and relationships of immune cells in the CNS.

Authors	Immunocyte	Role	Content	References
Alliot et al.	Microglia	Microglia arise from the yolk sac and emerge during embryonic development, increasing in number during gestation and steadily increasing in number during the first two weeks postpartum, when about 95% of microglia are born	The origin and proliferation of microglia	(25)
Lawson et al.	Microglia	Microglia are abundant in the brain, but their distribution is not uniform. They are more distributed in hippocampus, olfactory skull, basal ganglia and substantia nigra, and less distributed in fiber tracts, cerebellum and most brain stem	Morphology and distribution of microglia	(26)
Devanney et al.	Microglia and macrophage	The M2 phenotype of macrophages is age-dependent and can play an anti-inflammatory role, and the M1 phenotype is enhanced phagocytosis, but the M1M2 classification has limitations	The role of immune metabolism in neurotrauma	(27)
Gensel et al.	Macrophage	M1, M2a, M2b, and M2c macrophages were sequentially activated during the healing phase, but the duration of this phase was prolonged after the onset of injury inflammation	Role of macrophages in spinal cord injury	(28)
Tang et al.	Microglia	M1 microglia dominate the injury site at the end stage of the disease. At this time, the immunolysis and repair process of M2 microglia are inhibited, and endogenous stimulation may continue to activate M1 proinflammatory response, eventually leading to irreversible neuronal loss	Microglia can be classified into two phenotypes, M1/M2, and this classification has important roles in neurological diseases	(29)
Mills et al.	Macrophage	Lymphocyte Activation Macrophage Activation	The division of two phenotypes and paradigms of macrophages and their association	(30)
Wright-Jin et al.	Microglia	Microglia differentiate and remove brain inflammation to maintain CNS homeostasis	Role of microglia in CNS homeostasis	(31)
Takahashi et al.	NK	NK cells may actively inhibit potentially pathogenic autoimmune T cells that may mediate CNS inflammation	Role of NK in multiple sclerosis	(32)
Nimmerjahn et al.	Microglia	Microglia are highly active in their putative resting state, continuously investigate their microenvironment, and have extremely strong motility processes and protrusions	Highly dynamic monitoring of brain parenchyma by microglia *in vivo*	(33)
Miron et al.	M2 Microglia	The dominant response of microglia and peripheral macrophages changed from M1- to M2. M2 cell density is increased in lesions in aged mice	M2 cell polarization is essential for effective myelin regeneration and contributes to the treatment of multiple cell sclerosis	(34)
Boudreau et al.	NK	NK cells achieve structural diversity through mutation, making them specific and adaptive to different immune environments	Diversity in NK cell reactive capacities driven by NK education protect some individuals against a variety of infections and diseases	(35)
Bluestone et al.	T cells	Cytokines are important factors driving the differentiation of CD4 effector T cells	CD4 T cells are divided into functional subsets with different immune functions	(36)
Engelhardt et al.	T cells	Leukocytes cross the CNS barrier in response to chemokines and activators	During inflammation, T cells migrate across the CNS barrier and transport signals	(37)
Constant et al.	B cells	In the experiments designed to determine the ability of splenic dendritic cells (DCs) and B lymphocytes to take up peptide or protein Ags *in vivo*, Ags were taken up preferentially by DCs, whereas proteins were taken up by Ag-specific B cells *in vivo*	By presenting antigens to T cells, B cells cause T cells to perform protein antigens	(38)
Finkelman et al.	B cells	By activating B cells and presenting B cell antigens, anti-IGD antibodies induce T cell activation and tolerance	The antigen presentation of B cells is required to produce T-cell dependent antibody responses *in vivo*, and the antigen presentation of repetitive B cells to T cells is a necessary condition for the expansion of B cells.	(39)
Pistoia et al.	B cells	B cells can not only produce antibodies, but also present antigens to T cells	B cell function and its relevance to disease	(40)
Kipnis et al.	T cells	Autoimmune T cells can produce neurotrophic factors when activated by relevant antigens, and T cells can also activate non-immune cell colonization and participate in homeostasis restoration regulation	T cells and the immune system in schizophrenia	(41)

CNS, Central Nervous System.

Microglia are the main cellular component of the innate immune system of the brain. They are distributed in the whole brain at different densities, accounting for 5%-20% of the total brain cells, and are developed from primitive macrophages (25, 26). Microglia are activated and polarized after being exposed to external stimuli (42), which will present two phenotypes M1/M2 with different functional status and markers (27). The M1 phenotype produced by the general activation pathway can exert pro-inflammatory and neurotoxic effects, while the M2 phenotype produced by the selective activation pathway can exert anti-inflammatory and neuroprotective effects (28, 29), promoting medullary regeneration by inhibiting cell differentiation (34). Such a classification of microglia was originally proposed by Mills et al. (30). However, this classification method based on stimuli does not reflect the range and function of phenotypes well, and Mills himself has also shown that the main existence should be a continuous intermediate of M1 and M2 (43), so this once widely used classification method has been questioned by many people (44, 45). Later, Devanney et al. proposed a function-based classification method (27). For the function of microglia, there has been a high similarity between microglia and macrophages due to their structural characteristics (46), researchers have long thought that its function is mainly an immune response, like macrophages, to recognize and take up pathogens and other substances, and analyze their environment (33). However, many subsequent experiments have shown that, especially when there is no need for immunity, the role of microglia is mainly to carry out sensing, information processing and nerve protection in the central nervous system (25), and maintain the homeostasis of the central nervous system (31, 47). It promotes neural development over a period of time (48) and can affect nerve impulses in the adult brain (49).

Lymphocytes are the main cells of the body’s immune response, which are produced by lymphoid organs and can respond to signals such as foreign pathogens or inflammatory stimuli. T cells, B cells and natural killer cells play a significant role in central nervous system immunity. The main reason for the suppression of immune action in the CNS is the blockage of the blood-brain barrier (50, 51). Only a small number of lymphocytes can enter the CNS through the blood-brain barrier, blood-meningeal barrier, and blood-cerebrospinal fluid barrier during brain health and injury, and play an immune function or promote neuroinflammation (52–54).

T cells and B cells together constitute acquired immunity. They can all differentiate into different subtypes (55), release different cytokines or perform different functions, which greatly contributes to their immune specificity (36, 40, 56–58).

B cells travel from the skull to the meninges through blood vessels (59, 60), and their main role is to produce antibodies (61). After receiving antigen presentation, B cells can differentiate into plasma cells and memory cells. The former will produce specific antibodies that bind to the antigen and destroy it[98][99]. B cells can also act as antigen-presenting cells (APCs), processing antigens and presenting them to antigen-specific T cells (38, 39, 62), and promote the development of pro-inflammatory T cells (63). In the treatment of CNS autoimmune diseases, by depleting activated B cells, B cells as APC can be stimulated to target T cells, so that they can differentiate and develop, thus enhancing the immune effect (64, 65).

T lymphocytes, the sentinels of the adaptive immune system, respond to antigen-specific signals by bursting, proliferating, and differentiating into effector subsets to recognize and eliminate threats to the host (66, 67). In addition to the previously mentioned antigen presentation and immune role with B cells, T cells also have the function of promoting cognitive learning in the brain (41, 68, 69), as well as a certain role in maintaining CNS homeostasis. A large number of T cells are used for immune monitoring of the brain barrier (37).

Natural killer immune cell (NK) is a kind of large granular hematopoietic cell derived from bone marrow (70), and its function varies in different individuals due to different genes (35). Like T and B cells, NK also has a variety of subtypes (71), among which the CD56 bright NK cells are the main ones present in CD56 bright NK cells (72). It has been suggested that NK may play a regulatory role in central nervous system diseases (32). NK can repair nerves after CNS damage, coordinate immune responses, and inhibit the development of inflammation (73, 74). It is able to regulate the neurological diseases such as Parkinson disease, and can play a neuroprotective role by killing T cells that promote neurotoxicity (75). In some neuroinflammation, such as autoimmune encephalomyelitis (EAE), the cytotoxicity is reduced and the memory EAE is inhibited (76, 77). In addition, NK can also work with other lymphocytes (78) to cooperate in immune action in neurological diseases such as Alzheimer’s disease (79).

Recent studies have shown that astrocytes play an important role in CNS immunity by associating with other immune cells. For example, the recognition of astrocytes depends on microglia, and the subsequent release of caspases induced by astrocytes promotes apoptosis and reduces cellular inflammation. IF-33 released by astrocytes can recruit twice as many T cells and promote the repair of CNS diseases such as neurodegenerative diseases and spinal cord injuries (80).

### Immune system

The CNS immune system is divided into innate immunity and specific immunity, which make different immune responses in different situations. Some autoimmunity can lead to diseases, but the immune effect after injury helps to recover from injury (81).

After CNS injury, a series of subsequent immune responses are triggered, first innate immunity and then specific immunity. Both types of immunity can expand their number by releasing cytokines and regulating genes from immune cells (82–84). The innate immune system is the first line of defense to help the central nervous system resist foreign pathogens, and the specific immune response is slow, which will gather and play a role after inflammation or injury occurs (85, 86). A correct understanding of the immune system in different CNS diseases at different stages of development can help us find appropriate immunotherapy methods.

Due to the particularity of the CNS, the BBB still acts as a barrier, making the CNS an immune privileged organ. Its carrier and receptor proteins are the only way for outside proteins to enter the CNS, ensuring precise regulation of what enters the brain (87). This barrier creates an important difference between CNS immunity and peripheral nervous system immunity. There are only a small number of protoimmune cells in the CNS. These immune cells and some immune progenitors release chemokines and cytokines to recruit peripheral immune cells after brain injury, thus amplifying the immune effect (88). For example, fatty acids can activate peripheral macrophages through TLR4-mediated mechanism. Such recruitment needs to be carefully regulated, and the process will be discussed later (89).

However, the CNS has connections to all surrounding organs (90). There is an external barrier to immune cells, but the immune system of CNS is not isolated. Cerebrospinal fluid is used as the medium to exchange immune cells with the outside world through the meninges. Compartmentalized immune cells are recruited to cross the blood-brain barrier when the brain needs immune repair (91). These barriers used to be thought to insulate the CNS from its immune system, but recent research suggests that these barriers facilitate communication between the CNS and the outside world. Some of them also act as immune hubs, such as the dura mater, which associates with the brain through the lymphatic system, metabolizing waste and maintaining brain homeostasis (92).

## CNS disease and the association with immune system

Central nervous system diseases include central neurodegenerative diseases, nervous system infections, brain tumors and other types of diseases, including brain injury, spinal cord injury and peripheral nerve injury from the point of view of injury and disease location. These diseases are related to the immune system to some extent. Here we focus on central neurodegenerative neurological diseases, CNS tumors, traumatic brain injury, and spinal cord injury.

### CNS neoplasms

Brain tumors are generally divided into two categories, namely those from external sources, also known as metastases, and those arising from the central nervous system, such as gliomas (93). We focus on tumors arising spontaneously in the central nervous system. When tumors occur, tumor associated macrophages (TAMs) are the main immune cells in the tumor microenvironment (TME) in the CNS. Studies have shown that the incidence of some primary tumors is very low after immunosuppression (94, 95), which indicates that TAM targeted inhibition plays an important role in tumor therapy (96). Take glioma as an example, it is a tumor developed by glial cells, among which glioblastoma (GBM) is the most common glial tumor and one of the highest mortality among all cancers (97). The average overall survival time is not more than 15 years, and only seven percent of patients can be cured (98, 99). Some other subgroups of low-grade gliomas have molecular features similar to glioblastoma, with an invasive process (100). Glial cells will release CSF-1 and other cytokines and recruit TAM (101). Studies have shown that TAM can promote the growth rate and morphological size of glioma cells (102, 103), and a small number of TAMs and microglia will lead to a larger volume of tumors (104). In fact, in GBM, the number of TAMs is positively correlated with tumor severity, and there are very few in patients who do not relapse after recovery (105). According to this characteristic of TAM, the development of tumor therapy targeting TAM has become a new direction for brain tumor treatment (106, 107).

CNS tumor immune process after the basic follow - tumor immune system cycle, this is Chen et al. Applied to inducing tumor immune response rule (108), after the tumor cells results in the release, antigen precursor release activating factor to activate T cells, as effector cells infiltrating tumor, T cell contact and identify the antibody, to destroy, then the immune cells apoptosis (109). T cells can track and eliminate cancer by recognizing specific exogenous factors to track and damage host cells (79). In fact, the development of tumor immunotherapy based on this principle has been widely carried out and achieved remarkable results (110, 111), and the application in brain tumors is also an example to follow.

### Degenerative neurological diseases

In 2019, there were about 50 million people living with neurodegenerative diseases worldwide, and this number is expected to rise to 152 million by 2060 (112). In his study, Richard Armstrong listed a series of factors that lead to degenerative neurological diseases and pointed out that age is the most important risk factor (113). The two diseases with the highest incidence of degenerative neurological diseases are Alzheimer’s disease (AD) (114) and Parkinson’s disease (PD) (115, 116), which are also a hot research topic in CNS diseases.

An important component of neurodegenerative diseases is the triggering of innate immune mechanisms. Microglia and other cell types in the brain can be activated by misfolded proteins or abnormally localized nucleic acids. This detents microglia from their physiological and beneficial functions and leads to a sustained release of their proinflammatory mediators (25). In the process of Parkinson’s disease, for example, after the activation of microglia as the main cells of immune function, may cause nerve nutrition through compound, such as brain derived neurotrophic factor, nerve protective effect, but there are also likely to produce neurotoxicity of proinflammatory cytokines (such as tumor necrosis factor (TNF), interleukins) (117, 118). For these two functions, which are almost opposite to the development of the disease, the current view is that at the beginning, the cytokines of activated microglia may have neuroprotective effects, but then the activated microglia undergo toxic degeneration, leading to the progression of Parkinson’s disease to neurotoxicity (119). Sawada showed in his experimental studies that activated microglia may be neuroprotective in neonatal mice but neurotoxic in aged mice. This conclusion is consistent with the fact that age is a major risk factor in the pathogenesis of degenerative neurological diseases, because the performance of two subsets of activated microglia (with toxic and neuroprotective functions, respectively) is strongly correlated with age.

### Spinal cord injury

Spinal cord injury (SCI) refers to the injury of the spinal cord and the pathological changes such as sensory and motor disorders after the body encounters direct or indirect external interference. Spinal cord injury may lead to loss of limb perception, incontinence and even complete paralysis, which may not recover for a lifetime or even be life-threatening (120).

After SCI, to ensure the regeneration of damaged axons (121), the CNS begins to recruit peripheral neutrophils into the CNS (122) and begins to clear axons and myelin debris nerve remnants (123), within the first hour after injury, playing a major clearing role. Subsequently, the recruited neutrophils begin to apoptosis and release attractant factors that attract and recruit monocytes, and at the same time can also recruit certain macrophages. After reaching the injury site, monocytes differentiate into macrophages according to the chemokines at the injury site (124–126), and the macrophages produced at this time will phagocytize the apoptotic neutrophils and other tissue debris (127). Similarly, microglia are also involved in injury repair (128). So similar to the white blood cells, neutrophils and other related immune cells in the spinal cord damage happens after apoptosis, part of the nerve ending structure will be destroyed in the process of damage (120). Experiments have shown that there are many repair factors in the central nervous system that promote regeneration of biological factors in nerve and immune cells (Ramer, Ramer et al., 2005), but regeneration does not represent full functional recovery. Therefore, after the immune system deals with spinal cord injury, the body will have reduced immunity and be prone to infection (129). Moreover, although the immune system responds to traumatic stimuli, it drastically changes as the injury worsens, which may exacerbate the injury and inflammation (130, 131).

### Traumatic brain injury

Traumatic brain injury (TBI) is an injury to the brain caused by external forces or external shocks, which may lead to a reduction or change in the state of consciousness and easily cause biochemical cascade damage, so it may be accompanied by long-term sequelae (132). After the occurrence of TBI, symptoms such as intracranial hemorrhage, brain contusion, concussion, and damage to nerve synapses are first caused (133), and a cascade of damage occurs within minutes to months. TBI has a high incidence among both military personnel and the general public (134).

Neuroinflammation and peripheral neuropathy are easily triggered after TBI (135). There are many factors that cause this situation, such as purines, heat shock proteins, and receptors of pathogen associated endogenous molecules (PAMPs) and damage associated endogenous molecules (DAMPs), which may promote inflammation (136–138). The latter can combine with proteins to form inflammasomes, release and infiltrate microglia, astrocytes and other cell populations, and produce proinflammatory factors after activation (139). Proinflammatory factors can further lead to neuroinflammation, and more severe inflammation can even transform TBI into chronic neurological diseases (140).

Similar to other neurological diseases, in TBI, immune cells can both promote recovery and aggravate injury (141). TBI can instantly induce cell death, after which the damaged cells release DAMPs, signal to immune cells, recruit microglia, astrocytes, etc. After they are activated, they will further recruit peripheral immune cells to pass through the damaged blood-brain barrier (142). Microglia respond immediately after injury and accumulate in large numbers in the injured area (33, 143), removing cell debris through phagocytosis (44). It can also release trophic factors to protect nerves (133) and is involved in remodeling injured nerves (144). However, microglial activation will produce excessive inflammatory mediators, recruit peripheral immune cells, produce a large number of pro-inflammatory factors and cytotoxic substances, hinder the repair of the central nervous system, and even lead to cell death and neuronal dysfunction (145, 146). Similarly, although neutrophils can participate in the immune regulation of TBI, they will also release some acute inflammatory cytokines to aggravate brain injury. The protective and injury effects largely depend on the location, type and stage of injury (147). Which more complicated is that the immune function and immune effect after TBI are related to gender (148) and age (149). Therefore, studying the relationship between these influencing factors and immune effect is an important entry point for the treatment of TBI (**Table 2**).

**Table 2:** Studies on the role of different immune cells in TBI.

Authors	Immunocyte	Role	The research methods	References	
Roth et al.	Microglia, astrocytes	The skull is permeable to small molecular weight compounds and uses this delivery route to modulate inflammation and therapeutically ameliorate brain injury through transcranial administration of the ROS scavor glutathione	Traumatic Head Injury Neuroimaging Classification (THINC)	(133)	
Davalos et al.	Microglia	ATP is an important signaling molecule that mediates interactions between various cell types in the brain, and glial and endothelial cells may contribute to microglial responses by releasing large amounts of ATP upon injury	Microglia were imaged *in vivo* using a two-photon microscope	(137)	
Liu et al.	Microglia, astrocytes	The innate immune system engages in a series of PRRS to detect “danger” signals, such as PAMPs or DAMPs, to defend against infection or injury. NLR recognizes many PAMPs as well as various DAMPs to activate the assembly of inflammasomes that trigger the maturation of proinflammatory cytokines, such as IL-1β and IL-18	Western blot analysis	(139)	
Liu et al.	Neutrophils	Neutrophils are an important component of the innate immune system, and their inappropriate or excessive activation may lead to tissue damage.	Drug blockade, etc.	(147)
Damani et al.	Microglia	Most microglia are long-lived cells that have a long residence time in the CNS and are therefore susceptible to the *in situ* aging effects that occur during the normal lifespan of the animal	*In vitro* imaging of the explant retina	(149)	
Doering et al.	Zinc-rich (ZEN) neurons	Zn ions are protective against TBI effects	Diseased mice were treated with DEDTC or selenite	(150)	
Loane et al.	Microglia	In injured brains, microglia produce neuroprotective factors that remove cellular debris and coordinate the process of nerve repair	Drug therapy, gene blocking, etc	(142)	
Griffin et al.	Microglia-directed neurons	Traumatic brain injury, mild and severe, open and closed, leads to immune suppression and infection	Autologous reinfusion of WBCs (adoptive immune therapy)	(132)	
Jassam et al.	Microglia, astrocytes	TBI induces an immune response composed of locally and peripherally derived participants that begins within minutes of the onset of TBI if the injury does not resolve or causes chronic diseases such as chronic traumatic encephalopathy	Inhibitors block immune cell activation	(44)	
Edwards et al.	Steroids	Refutes any substantial reduction in corticosteroid mortality or severe disability within 6 months after traumatic brain injury	MRC CRASH: A randomized controlled trial of the effects of corticosteroids	(141)	

TBI, Traumatic brain injury.

Now the treatment of some kinds of new ideas are mainly concentrated in cell therapy (151), because the immune cells such as macrophages have different phenotypes, their functions have significant difference, so can adjust the balance between different phenotypes, as far as possible avoid inflammation cause of neurodegenerative diseases, lower immune cell toxicity effect (152). In a study of the effects of Anakinra, a recombinant human IL-1 receptor antagonist, the treatment group had a higher cure rate than the control group (153). In addition, some small particles, such as zinc ions, have also been shown to be involved in neuroprotection and nerve recovery after injury (150). TBI ZnT3-KO mice were more severely injured when compared with juvenile wild-type mice.

**Figure 2:**
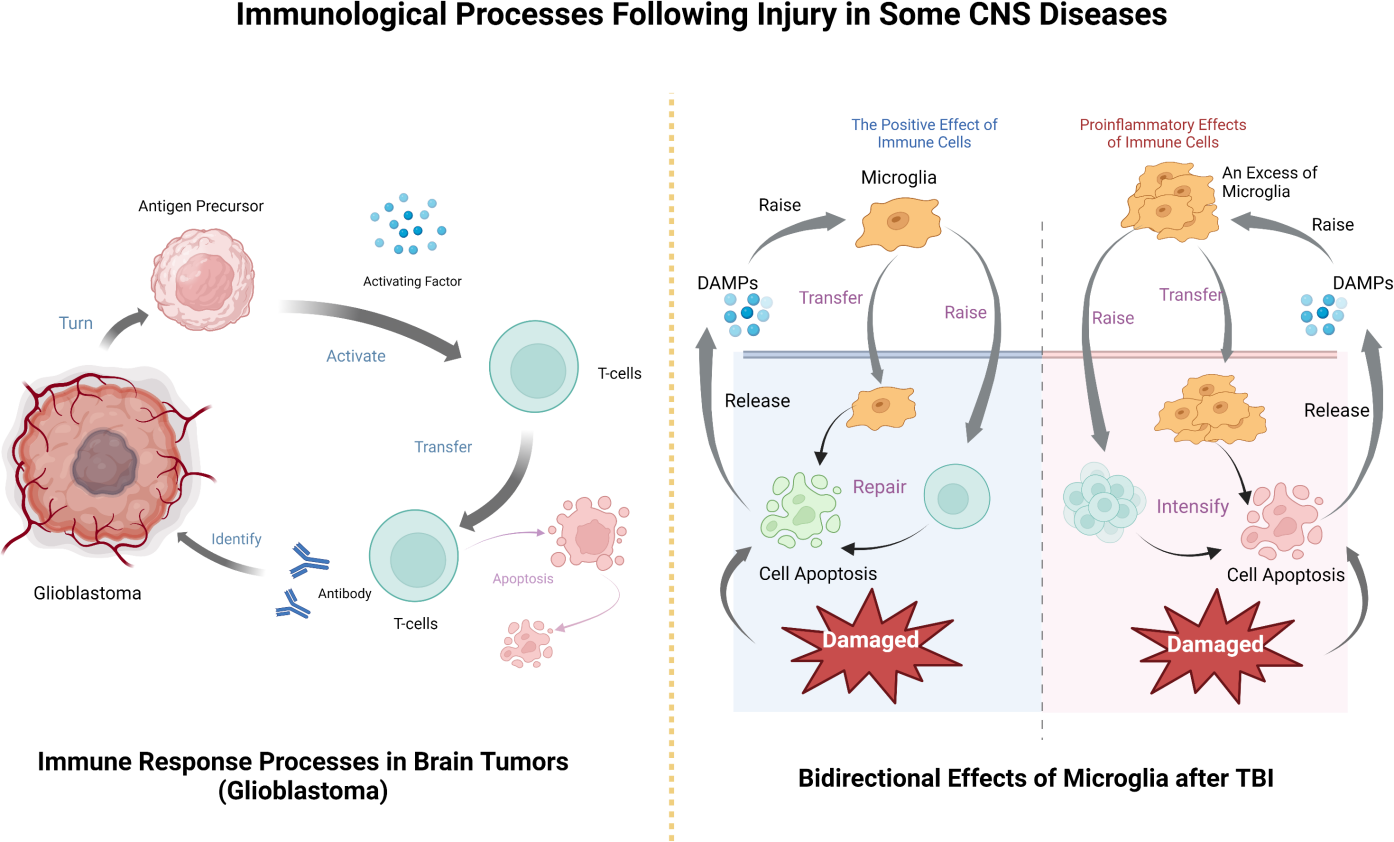
The processes by which immune cells exert their effects after CNS tumors and TBI, including their bidirectional effects on the disease.

In each of these diseases, the brain is damaged to a degree that releases specific substances that activate the innate immune system, which in turn activates and recruits the peripheral immune system. The immune system plays an important role in the anti-inflammatory response to disease injury and subsequent damage repair, but some immune responses also lead to the deterioration of the disease, so it is believed that CNS diseases and CNS immune system have an important relationship (**Figure 2**).

## Conclusion and future prospects

Although some immune cells in the CNS have been discovered and their functions have been relatively obvious, a large part of the CNS is still unknown. Previous studies have shown that the molecular pattern and molecular reason for these apparent phenomena are still unclear after addressing the expression role of the immune system. For example, do NK cells maintain or regulate repair functions in the central nervous system and how do they do it? What is the mechanism of macrophage phagocytosis and foam cell formation after SCI? How do macrophages recognize and internalize specific molecules in apoptotic neutrophils? All these problems now seem difficult to solve (154, 155).

In addition, some immune cells were surprisingly found to be present in specific disease contexts, which could not be explained by current understanding. Some CNS diseases currently have no suitable treatment (156, 157), and some therapies that appear to be effective have substantial limitations. For example, although checkpoint inhibitors have been relatively successful in many solid tumor types, they have been difficult to succeed in CNS tumors. Even some serious CNS diseases have not received enough attention (158).

In the future studies, more neurological diseases will be taken into account, and the immune connection between the CNS and the peripheral nervous system may become an important consideration of immune effects.

## Author contributions

JX and CM had the idea for the article. MH and JL performed the literature search and data analysis. JW and ZX drafted and critically revised the work. All authors contributed to the article and approved the submitted version.

## Conflict of interest

The authors declare that the research was conducted in the absence of any commercial or financial relationships that could be construed as a potential conflict of interest.

## Publisher’s note

All claims expressed in this article are solely those of the authors and do not necessarily represent those of their affiliated organizations, or those of the publisher, the editors and the reviewers. Any product that may be evaluated in this article, or claim that may be made by its manufacturer, is not guaranteed or endorsed by the publisher.
